# Circulating pTau181 and Genomic Background in Dogs: A Pilot Study on Neurobiological Aging

**DOI:** 10.1155/vmi/7159552

**Published:** 2026-08-30

**Authors:** Valentina Gazzano, Maria Chiara Fabbri, Simona Capsoni, Carlo Cantile, Lara Tinacci, Angelo Gazzano, Maria Claudia Curadi, Francesca Cecchi

**Affiliations:** ^1^ Department of Veterinary Science, University of Pisa, Viale delle Piagge 2, 56124, Pisa, Italy, unipi.it; ^2^ Department of Agriculture, Food, Environment, and Forestry (DAGRI), University of Florence, Florence 50144, Italy, unifi.it; ^3^ Department Neuroscience & Rehabilitation, University of Ferrara, 44124, Ferrara, Italy, unife.it; ^4^ Laboratory of Biology, Scuola Normale Superiore, 56127, Pisa, Italy, sns.it

**Keywords:** dog aging, genomic inbreeding, GWAS, Labrador Retriever, phosphorylated tau

## Abstract

Aging in dogs mirrors key biological aspects of human aging, including molecular processes associated with neurodegeneration, making the dog a valuable comparative model. This pilot study investigated the integration of circulating blood biomarkers and genomic architecture to explore interindividual variability associated with neurobiological aging in dogs. Forty‐eight dogs (31 purebred Labrador Retrievers and 17 mixed‐breed dogs; age range: 2–15 years) were analyzed for serum phosphorylated tau at threonine 181 (pTau181). Genome‐wide SNP genotyping was performed to characterize population structure, estimate pedigree‐ and ROH‐based genomic inbreeding, and conduct an exploratory genome‐wide association study (GWAS). Circulating pTau181 produced a quantifiable signal, but a subset of values fell below the assay’s lower limit of detection and were coded as zero for statistical analysis. Data showed a continuous distribution, with mean values ranging from 6.1 to 13.4 pg/mL across demographic groups. No significant associations were observed between pTau181 concentrations and age category, sex, or breed group. Genomic analyses revealed clear population stratification between Labrador Retrievers and mixed‐breed dogs, with significantly higher genomic homozygosity in Labradors (FROH = 0.26 ± 0.05) compared with mixed breeds (FROH = 0.12 ± 0.14; *p* = 0.00025). No significant correlations were detected between circulating pTau181 levels and genomic inbreeding coefficients. Exploratory GWAS identified a suggestive association between pTau181 concentrations and a locus on chromosome 29 (top SNP *p* = 5.57 × 10^−7^), encompassing genes involved in proteostasis, neurodevelopment, and cellular metabolism. Although these associations did not reach genome‐wide significance, they point to a biologically coherent genomic region potentially contributing to interindividual variability in tau blood levels. Overall, these findings provide preliminary evidence supporting the feasibility of combining circulating pTau181 measurements with genome‐wide analyses to investigate molecular and genetic aspects of neurobiological aging in dogs within a comparative research framework.

## 1. Introduction

The progressive increase in life expectancy observed in companion animals has led to a growing prevalence of age‐related biological research, including molecular processes associated with neurodegeneration [1, 2].

Among companion species, the dog represents a particularly valuable natural model for the study of brain aging, as it shares with humans not only a prolonged lifespan and comparable environmental exposures but also the spontaneous emergence of aging‐related neurobiological and cognitive alterations [3–7].

Beyond phenotypic manifestations, individual variability in susceptibility to age‐related neurobiological changes is increasingly recognized as being modulated by genetic background. Genomic diversity, population structure, and inbreeding levels may influence neuronal resilience, molecular homeostasis, and vulnerability to age‐associated alterations in the nervous system [8, 9]. Understanding how genetic architecture interacts with molecular biomarkers of aging represents a key challenge in both veterinary and comparative neuroscience.

At the molecular level, physiological aging and neurodegenerative processes are characterized by progressive alterations in neuronal maintenance mechanisms, including cytoskeletal stability and protein homeostasis [10]. Among the proteins involved, tau, a microtubule‐associated protein, plays a central role in maintaining axonal structure and intracellular transport under physiological conditions [11]. Tau function is tightly regulated by posttranslational modifications, particularly phosphorylation, which modulates its affinity for microtubules and intracellular localization [12, 13].

During normal aging, tau undergoes region‐ and stage‐dependent changes in phosphorylation that may occur even in the absence of overt neurodegeneration, supporting the concept of a continuum between physiological brain aging and early pathological processes [14–17]. Pathological tau is characterized by aberrant hyperphosphorylation, conformational changes, and reduced microtubule binding, leading to tau mislocalization and aggregation into soluble oligomers and insoluble fibrillar structures that accumulate intracellularly as neurofibrillary tangles [11, 12, 18]. Importantly, tau pathology shows a strong association with synaptic dysfunction and neuronal loss, often correlating more closely with disease severity than other neuropathological features [19, 20].

Increasing evidence indicates that tau dysregulation may arise as a primary age‐related process and act as a driver of neurodegenerative vulnerability [17, 21, 22]. Conditions such as primary age‐related tauopathy highlight the frequent emergence of tau aggregation in the aging brain, even in the relative absence of other hallmark lesions [17, 22, 23]. Additional mechanisms, including impaired proteostasis, oxidative stress, and neuroinflammatory processes, may independently contribute to tau phosphorylation and neuronal dysfunction during aging [22, 24, 25].

From a biomarker perspective, phosphorylated tau species have emerged as sensitive indicators of tau‐related molecular alterations. In particular, tau phosphorylated at threonine 181 (pTau181) has gained prominence as a marker reflecting dynamic neurobiological changes rather than nonspecific neuronal damage, rising in the initial stages of the Alzheimer’s continuum even when other pathological hallmarks are only subtly detectable [26–28].

Circulating pTau181 can be measured in cerebrospinal fluid and blood using immunoassay‐based platforms [29, 30] and has been shown to increase early during aging‐related neurodegenerative processes, following trajectories that are partially dissociated from aggregated tau pathology [31–33]. In dogs, tau hyperphosphorylation has been documented in brain tissue in association with canine cognitive dysfunction and other neurological conditions [34, 35]. More directly relevant to the present study, phosphorylated tau species have also been measured in peripheral blood in dogs, including a preliminary report on circulating phospho‐tau and beta‐amyloid in relation to inbreeding in Labrador Retrievers [36]. However, evidence from human and murine transgenic models indicates that the relationship between peripheral tau measurements and central neurobiological processes remains incompletely understood [37, 38], and comparable data specifically addressing this relationship in dogs are currently lacking.

Beyond molecular mechanisms, genomic architecture plays a crucial role in shaping biological aging. In humans, genome‐wide association studies (GWASs) have demonstrated that neurodegeneration‐related traits are highly polygenic, involving pathways related to immune response, synaptic function, cellular metabolism, and protein turnover [39, 40]. In addition to specific risk loci, genome‐wide measures of homozygosity and relatedness have emerged as modifiers of vulnerability to age‐related neurological changes, suggesting that reduced genetic diversity may compromise neuroprotective mechanisms [41–44].

Domestic dogs provide a unique comparative framework for investigating the biological consequences of genetic diversity and inbreeding [45]. Artificial selection, breed formation, and closed breeding populations have generated pronounced heterogeneity in genomic architecture across breeds, resulting in wide variation in linkage disequilibrium, runs of homozygosity (ROH), and pedigree‐based inbreeding levels [46–48]. These genomic features influence multiple health‐related traits, including lifespan and susceptibility to neurological conditions, making dogs an informative model for studying the interaction between genetic diversity and neurobiological aging [48].

Recent advances in canine genomics allow the quantification of individual inbreeding using complementary genome‐wide metrics that capture both recent and ancient autozygosity, beyond traditional pedigree‐based estimates [49]. However, the relationship between genomic inbreeding and circulating biomarkers associated with neurobiological aging remains largely unexplored in dogs.

Against this background, the present pilot study aimed to investigate circulating phosphorylated tau 181 as a potential peripheral marker of neurobiological aging in dogs and to explore its relationship with genomic architecture, including population structure, genomic inbreeding, and exploratory genome‐wide association signals, in purebred Labrador Retrievers and mixed‐breed dogs. As circulating pTau181 may be influenced by peripheral clearance and metabolic factors independent of central nervous system processes, its interpretation as a marker of neurobiological aging would ultimately require validation against neuropathological or neuroimaging endpoints, which was beyond the scope of the present pilot study.

## 2. Materials and Methods

### 2.1. Animal Sampling

A total of 48 dogs were included in this study, comprising 31 purebred Labrador Retrievers and 17 mixed‐breed dogs, with ages ranging from 2 to 15 years. To capture age‐related variation while ensuring balanced group sizes, dogs were assigned to two clinically relevant age categories: young (< 10 years) and senior (≥ 10 years). Purebred Labrador Retrievers were recruited from privately owned dogs presented to the Veterinary Teaching Hospital of San Piero a Grado (University of Pisa) for routine clinical evaluation or non‐neurological conditions. Mixed‐breed dogs were enrolled from two municipal animal shelters: the Pisa facility (“Soffio di Vento,” Via di Granuccio, 56121 Pisa, Italy) and the Lucca facility (“Pontetetto,” Via Santeschi, 993, 55100 Lucca, Italy). Both shelters are managed by the same nonprofit organization, the “Ponteverde” social cooperative (Piazza Vittime dei Lager Nazisti, 3, 56025 Pontedera, PI, Italy), and operate under comparable management routines and housing protocols. Dogs were enrolled based on availability during the study period and upon obtaining owner or institutional consent. All dogs underwent a general clinical examination at the time of sampling, and no overt behavioral or cognitive abnormalities were reported at enrollment; however, no validated cognitive assessment instrument was administered. Accordingly, the study population can be regarded as an opportunistic sample of clinically unremarkable dogs, with no intentional bias introduced for these variables, consistent with the exploratory design of the study.

All procedures adhered to current Italian animal welfare legislation and were performed as part of routine veterinary clinical practice. The study protocol was reviewed and approved by the University of Pisa Animal Ethics Committee as nonexperimental clinical practice (Delibera n. 51/2021, November 26, 2021).

### 2.2. Blood Collection and Biomarker Quantification

From each dog, a peripheral blood sample of 5 mL was collected by venipuncture. Blood samples were processed according to standard laboratory procedures to obtain serum, which was subsequently used for biomarker analyses.

Serum concentrations of phosphorylated tau at threonine 181 (pTau181) were quantified using a commercially available enzyme‐linked immunosorbent assay (ELISA) kit (Canine Phosphorylated Tau 181, Cat. No. MBS006469; MyBioSource, San Diego, CA, USA), following the manufacturer’s instructions. All samples were analyzed under identical experimental conditions.

### 2.3. Statistical Analysis of Phenotypic and Biomarker Data

Statistical analyses were performed using JMP Software Version 5.0 (SAS Institute Inc., Cary, NC, USA). Data normality was assessed using the Shapiro–Wilk test. Because pTau181 values included zero values and were not normally distributed, data were log‐transformed [ln (pTau181 + 1)] prior to analysis. Analyses of variance (ANOVA) were conducted with log‐transformed pTau181 as the dependent variable and breed, sex, and age category as fixed effects. Due to the limited sample size, interaction terms were not included. Statistical significance was set at *p* < 0.05.

### 2.4. DNA Extraction and SNP Genotyping

Genomic DNA was extracted from whole blood samples using the Monarch® Genomic DNA Purification Kit (New England Biolabs, Ipswich, MA, USA), following the manufacturer’s protocol for mammalian whole blood. Briefly, blood samples underwent enzymatic lysis and protein digestion, after which genomic DNA was purified using silica‐based spin columns. DNA was eluted in nuclease‐free buffer and stored at −20°C until analysis.

DNA quantity and quality were assessed spectrophotometrically using a NanoDrop™ One instrument (Thermo Fisher Scientific, Waltham, MA, USA) to verify suitability for downstream applications. All samples meeting quality requirements were subsequently subjected to genome‐wide SNP genotyping.

All enrolled dogs (*n* = 48) were genotyped using the Infinium CanineHTS‐24 BeadChip Kit (Illumina Inc., San Diego, CA, USA), which includes more than 170,000 single nucleotide polymorphisms mapped to the CanFam4.0 reference genome. SNP genotyping was outsourced to the Science and Technology Park of Sardinia (Porto Conte Ricerche, Alghero, Italy).

SNP quality control (QC) was performed with PLINK v.1.9 (Chang et al. 2015). Only autosomal SNPs with a call rate higher than 95%, a minor allele frequency (MAF) > 1%, and no extreme deviation from Hardy–Weinberg equilibrium (*p* value > 0.00001) were retained. Animals with more than 10% missing genotypes were discarded. After QC, 141,946 SNPs mapped to the 38 canine autosomes were retained for downstream analyses, comprising 17 mixed‐breed and 31 purebred individuals.

### 2.5. Pedigree‐Based Inbreeding Analysis

Pedigrees of purebred Labrador Retrievers were retrieved from the Italian Kennel Club (ENCI) database. A genealogical database was constructed comprising a whole population (WP), including all founders, ancestors, and descendants, and a reference population (RP), consisting of the 31 purebred Labrador dogs included in the study.

Individual and average pedigree‐based inbreeding coefficients (FPED) were calculated using CFC v1.0 [50], based on Colleau’s modified algorithm. FPED represents the probability that two alleles at a given locus are identical by descent [51], computed using the tabular method described by Meuwissen and Luo [52]. Inbreeding levels were categorized into four classes: *F* = 0; 0 < *F* ≤ 0.05; 0.05 < *F* ≤ 0.10; and 0.10 < *F* ≤ 0.15.

### 2.6. Population Structure Analysis

The architecture of Labrador and mixed breed populations was defined using three different approaches of population structure analyses: admixture, principal components analysis (PCA), and multidimensional scaling analysis (MDS). The number of ancestral populations was evaluated by admixture, implemented in the LEA R package. The cross‐entropy criterion was used to choose the number of ancestral populations and the best run for a fixed value of K (10 repetitions and *K* = 1, 2, 3 were run). Individuals with less than a 70% probability of belonging to the Labrador group were considered crossbreds. PCA, which produces orthogonal projections of the original data, was performed in PLINK v1.9 using –*pca* flag [53]. MDS analysis was also conducted in PLINK v1.9 with *–mds* flag based on the genome‐wide pairwise identity‐by‐state (IBS) distance matrix, and the first two dimensions were retained [53]. The results were visualized using the R package ggplot2 [54]. Together, these three methods provide complementary insights into population structure and were used to ensure the absence of major stratification that could bias downstream analyses.

### 2.7. GWAS and Gene Set Enrichment Analysis

A GWAS was performed using PLINK v1.9 [53]. After applying the same QC filters used for the PCA and additionally removing SNPs with extreme deviation from Hardy–Weinberg equilibrium (*p* < 1 × 10^−6^), 140,221 markers remained. Age, sex, and the first two principal components (PC1 and PC2) derived from the population structure analysis, capturing the primary axes of genomic variation between Labrador Retrievers and mixed‐breed dogs, were included as covariates to account for population stratification. A linear regression model (‐linear) was fitted for the Tau quantitative trait. Statistical significance was determined using a Bonferroni‐corrected threshold (adjusted *p* < 3.56 × 10^−7^; 0.05/140,221). Since the Bonferroni correction is highly conservative, markers with suggestive associations were determined using a threshold corresponding to one expected false positive per genome scan (adjusted *p* < 7.13 × 10^−6^; 1/140,221). Manhattan plot was created with the qqman R package [55].

Genomic windows of ± 250 kb around the significant SNPs were investigated to detect candidate genes using the Genome Data Viewer of NCBI (https://www.ncbi.nlm.nih.gov/gdv?org=canis-lupus-familiaris). For gene set enrichment analysis, lists of protein‐coding genes were uploaded to STRING v12 (https://string-db.org/cgi/input?sessionId=bc0hZVGxWJyG%26input_page_show_search=on).

### 2.8. ROH Analysis and Correlations Between Genomic Inbreeding and Biomarkers

Analysis of ROH was conducted with the R package detectRUNS v. 0.9.5 [56]. ROH were defined using the following criteria: (i) a minimum number of 50 consecutive SNPs; (ii) minimum ROH length of 1 Mbp; (iii) a maximum gap of 1 Mbp between consecutive homozygous SNPs; (iv) no opposite homozygous genotypes allowed; and (v) no missing genotypes permitted. The consecutive method was preferred over the sliding windows approach in order to avoid the detection of artificial ROH shorter than the window described above [57]. All ROH were classified into five length categories: 0–5, 5–10, 10–20, 20–40, and > 40 Mbp. For each group, the total number of ROH, the ROH average number per individual, the average length of ROH, the number of ROH per group per chromosome, and the number of ROH per length class were estimated. The genome‐wide between‐group ROH inbreeding (FROH) was compared using the Wilcoxon rank‐sum test.

Individual genomic inbreeding coefficients were calculated as described above for FROH. Additionally, *–het* and *–ibc* flags in PLINK 1.9 [53] were applied to calculate Fhet (observed and expected autosomal homozygous genotype counts), Fhat1 (the usual variance‐standardized relationship minus 1), Fhat2 (similar to the‐‐het estimate), and Fhat3 (based on the correlation between uniting gametes). Associations between all the aforementioned genomic inbreeding coefficients and biomarkers were evaluated using correlation analyses. Before analysis, Data normality was assessed with the Shapiro–Wilk test. Depending on data distribution, Spearman’s rank correlation coefficient (for nonnormally distributed variables) was applied. Statistical significance was set at *p* < 0.05. Correlations plots were generated using the corrplot R package [58].

## 3. Results

### 3.1. Blood Sampling

Table 1 summarizes the distribution of the sampled dogs according to breed (Labrador Retriever and mixed breed), sex, and age category (young and senior). Among Labrador Retrievers, females were more represented than males, with a total of 19 females and 12 males. Mixed‐breed dogs showed a more balanced sex distribution, with 9 males and 8 females overall.

**TABLE 1:** Distribution of animal samples by breed, sex, and age group.

Age group	Labrador		Mixed breed		
	Males	Females	Males	Females	Total
Young	3	5	6	4	18
Senior	9	14	3	4	30
Total	12	19	9	8	48

Both age categories were adequately represented in the two breed groups, with 18 young dogs and 30 senior dogs included in the study.

### 3.2. Circulating pTau181 Concentrations

Serum concentrations of pTau181 were evaluated according to breed group, sex, and age category. pTau181 produced a quantifiable signal, but values below the assay’s lower limit of detection were observed in a subset of samples and coded as zero for statistical analysis (Supporting Table 1).

Mean pTau181 concentrations were comparable across demographic categories, and no statistically significant differences were observed between Labrador Retrievers and mixed‐breed dogs, between males and females, or between young and senior dogs. A substantial overlap in pTau181 values was evident across all groups.

Among Labrador Retrievers, mean pTau181 concentrations ranged from 5.94 ± 1.79 pg/mL in young dogs to 10.91 ± 2.80 pg/mL in senior dogs (Table 2). Mixed‐breed dogs showed mean values ranging from 7.32 ± 3.19 pg/mL in senior dogs to 11.08 ± 4.84 pg/mL in young dogs. Individual pTau181 values spanned a wide range in both breed groups, indicating marked interindividual variability.

**TABLE 2:** Sample size, mean ± standard error, and minimum–maximum range of serum pTau181 concentrations (pg/mL) according to breed group, sex, and age category.

Group	Total, *n*; mean ± SE (range)	Males, *n*; mean ± SE (range)	Females, *n*; mean ± SE (range)	Young, *n*; mean ± SE (range)	Senior, *n*; mean ± SE (range)
Labrador Retriever	31; 9.63 ± 2.15 (0.00–37.72)	12; 9.35 ± 3.96 (0.00–37.72)	19; 9.81 ± 2.55 (0.00–36.97)	8; 5.94 ± 1.79 (0.00–13.76)	23; 10.91 ± 2.80 (0.00–37.72)
Mixed breed	17; 9.53 ± 3.09 (0.00–45.00)	9; 6.05 ± 2.76 (0.00–22.38)	8; 13.45 ± 5.69 (0.00–45.00)	10; 11.08 ± 4.84 (0.00–45.00)	7; 7.32 ± 3.19 (0.00–17.72)

### 3.3. Pedigree‐Based Inbreeding Analysis

Analysis of the pedigrees of the 31 purebred Labrador dogs (RP) shows that the complete database contains 343 dogs (WP). The population was stratified into seven traced generations.

Out of the 31 purebred animals, only 5 were found to be inbred (mean inbreeding coefficient *F* = 0.046; min = 0.002, max = 0.125), with one individual having *F* = 0.125 and the remaining four with *F* < 0.05. Given the small number of inbred individuals and the overall low inbreeding coefficients, it was not possible to reliably assess potential associations between inbreeding levels and pTau181 concentrations.

### 3.4. Population Structure Analysis

Population structure was assessed using three complementary approaches: ancestry inference (admixture), PCA, and MDS.

**FIGURE 1:**
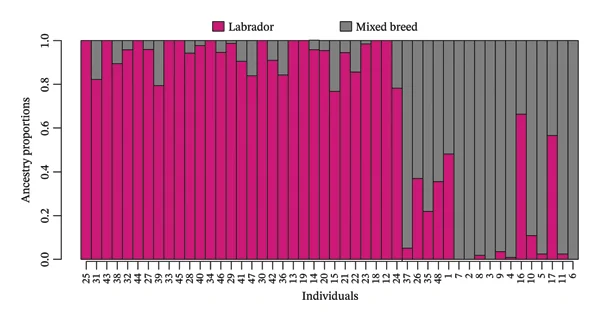
Proportion of ancestry for each individual inferred through admixture.

Figure 1 displays the ancestry proportions inferred for each individual. Consistent with the a priori breed classification, two well‐defined ancestral clusters were identified. Individuals with < 70% Labrador ancestry were classified as crossbred, in agreement with the admixture threshold commonly adopted in canine population genetics. This analysis confirmed the presence of two genetically distinct groups with minimal evidence of admixture leakage between them.

**FIGURE 2:**
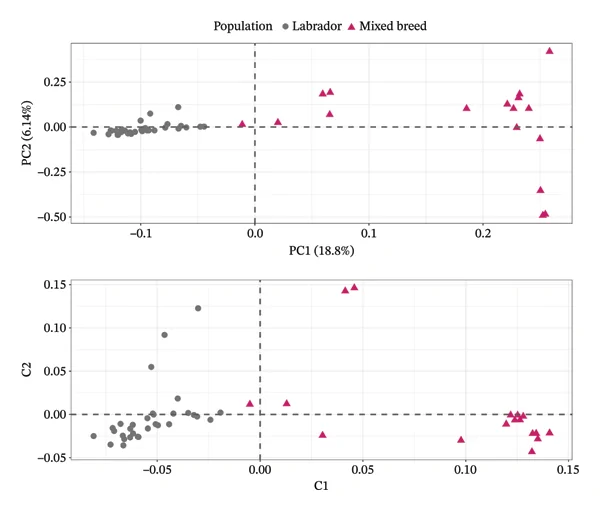
Principal component analysis and multidimensional scaling analysis of Labrador and mixed breeds populations.

PCA and MDS analyses further supported this clustering pattern (Figure 2). In both multivariate representations, Labradors formed a compact, homogeneous cluster, whereas mixed‐breed dogs were more dispersed, reflecting their heterogeneous genetic backgrounds. Notably, five crossbred individuals (IDs 16, 17, 26, 1, and 48) plotted near the Labrador group in both PCA and MDS space. These animals corresponded to individuals with intermediate Labrador ancestry (34%–65%), consistent with their genomic profiles and confirming the concordance among the three analytical approaches.

The overlap observed for these individuals suggests recent admixture or a higher proportion of Labrador ancestry in their genealogical background.

Overall, the combination of ancestry inference, PCA, and MDS reliably captured population structure without inconsistencies across methods, ensuring that subsequent association analyses (e.g., GWAS and ROH) were not confounded by misclassification or population misassignment.

### 3.5. GWAS and Gene Set Enrichment Analysis

**FIGURE 3:**
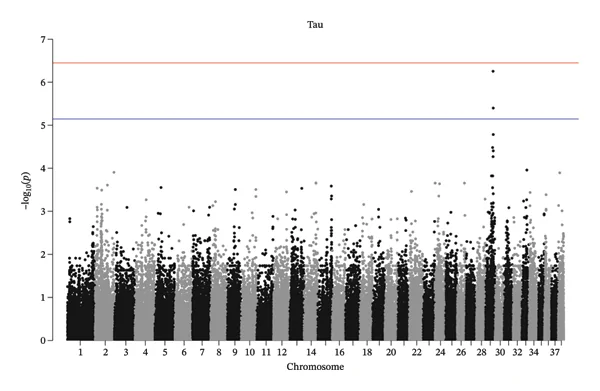
Manhattan plot of GWAS for Tau levels. The red line indicates the Bonferroni threshold; the blue line marks the suggestive significance threshold.

The GWAS conducted for Tau levels revealed two loci on chromosome 29 showing suggestive statistical associations (Figure 3). The most significant marker was BICF2S23418606 (p = 5.57 × 10^−7^, located at 33,176,691 bp), followed by BICF2G630632703 (p = 3.98 × 10^−6^, located at 33,500,707 bp). Although these SNPs did not surpass the genome‐wide Bonferroni threshold, their *p* values fall within the accepted range for suggestive associations in GWAS, warranting further investigation, especially due to the clear peaks in those regions (Figure 3). Candidate genes were identified within ± 250 kb of these SNPs based on the CanFam4.0 genome assembly.

The window around BICF2S23418606 (32,926,691–33,426,691 bp) contained three annotated protein‐coding genes of interest: PSKH2, ATP6V0D2, and SLC7A13, together with several uncharacterized loci. The window around BICF2G630632703 (33,250,707–33,750,707 bp) encompassed the annotated gene CNBD1 along with other predicted loci. Importantly, four genes (WWP1, RMDN1, CPNE3, and CNGB3) were shared between both intervals due to partial overlap of the two genomic windows. The full list of candidate genes is provided in Table 3.

**TABLE 3:** List of candidate genes located within the genomic windows (±250 kb upstream and downstream of the top two SNPs).

Start	Stop	Gene symbol	Related SNP
32,940,581	32,960,636	PSKH2	BICF2S23418606
32,971,567	32,982,132	LOC111093236	BICF2S23418606
32,980,580	33,022,805	ATP6V0D2	BICF2S23418606
33,029,082	33,029,216	LOC119866787	BICF2S23418606
33,060,731	33,078,247	SLC7A13	BICF2S23418606
33,126,990	33,129,744	LOC106558014	BICF2S23418606
33,147,532	33,269,942	WWP1	both
33,168,790	33,199,473	LOC106558016	BICF2S23418606
33,225,305	33,225,784	LOC102154790	BICF2S23418606
33,262,083	33,262,179	LOC119866808	both
33,267,771	33,329,421	RMDN1	both
33,298,344	33,299,568	LOC100855445	both
33,325,872	33,381,277	CPNE3	both
33,393,790	33,536,962	CNGB3	both
33,404,960	33,409,862	LOC119866739	both
33,503,458	33,506,128	LOC119866738	BICF2G630632703
33,636,794	34,168,638	CNBD1	BICF2G630632703
33,648,356	33,674,890	LOC111093149	BICF2G630632703
33,700,386	33,701,024	LOC608680	BICF2G630632703

**FIGURE 4:**
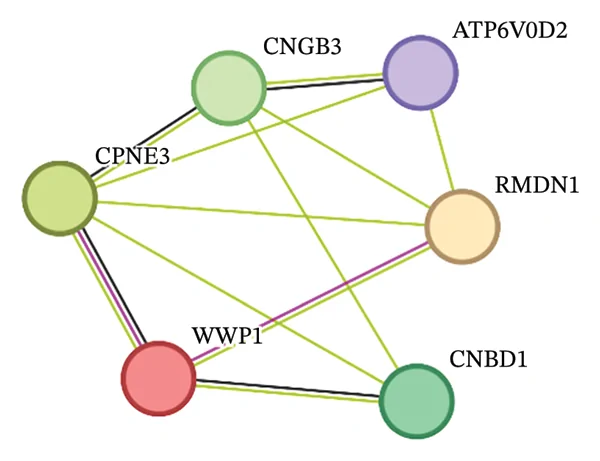
Cluster identified among the genes within the significant genomic windows.

STRING functional network analysis (Figure 4) revealed a highly interconnected cluster comprising six of the eight annotated genes (CNGB3, CPNE3, WWP1, CNBD1, RMDN1, and ATP6V0D2). The presence of a single, strongly connected module suggests potential coregulation or shared biological pathways relevant for neurodegeneration‐related traits, although functional validation is required.

Together, these results highlight a small but biologically cohesive chromosomal region that may modulate circulating Tau levels in dogs, supporting the rationale for further targeted sequencing or validation in larger cohorts.

### 3.6. ROH Analyses and Correlations Between Genomic Inbreeding and Biomarkers

A total of 6142 ROH were detected across all individuals, with substantial differences between groups: 4871 ROH in Labradors and 1271 ROH in mixed‐breed.

The average number of ROH per individual was 157 ± 12 in Labradors and 75 ± 46 in mixed‐breed dogs. Labrador Retrievers exhibited a markedly higher average number of ROH per individual than mixed‐breed dogs, consistent with the closed breeding structure and smaller effective population size typical of purebred populations.

Consistent with this interpretation, the mean ROH length was also slightly higher in Labradors (3.63 Mb) than in mixed‐breed dogs (3.43 Mb), further supporting more pronounced recent autozygosity in the purebred group. Together with the higher genomic inbreeding (FROH) observed in Labradors relative to mixed‐breed dogs, these findings converge to indicate a consistently higher degree of genomic homozygosity across multiple complementary metrics in the closed purebred population, whereas the comparatively high total ROH count in mixed‐breed dogs despite their lower per‐individual average and lower FROH likely reflects a larger number of short, ancestrally shared haplotype segments distributed across a genetically more heterogeneous group, rather than recent inbreeding.

ROH were classified into five length categories (0–5, 5–10, 10–20, 20–40, and > 40 Mb) to characterize the distribution of homozygosity. The 0–5 Mb class was the most abundant in both groups, particularly in Labradors (Table 4). Longer ROH (> 20 Mb), typical signatures of recent inbreeding, were more frequent in the Labrador group.

**TABLE 4:** Number of runs of homozygosity for length classes (Mb).

Class	Mixed breed	Labrador
0–5	1058	3874
05–10	120	626
10–20	68	305
20–40	22	61
< 40	3	5

**FIGURE 5:**
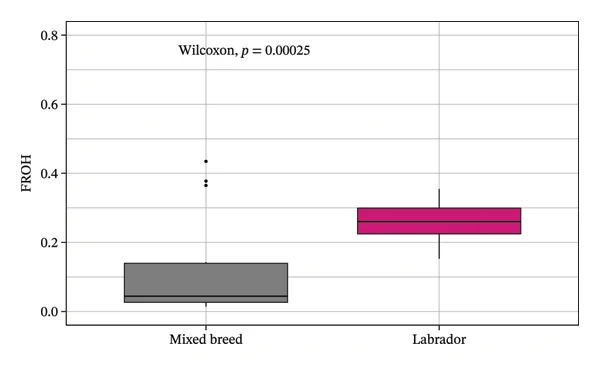
Runs of Homozygosity inbreeding (FROH) distribution in Labrador and mixed‐breed populations with Wilcoxon *p* value.

The genome‐wide inbreeding coefficient based on ROH (FROH) differed markedly between groups, with Labradors showing significantly higher values (0.26 ± 0.05) than mixed breeds (0.12 ± 0.14). A Wilcoxon rank‐sum test confirmed that this difference was statistically significant (*p* = 0.00025; Figure 5).

**FIGURE 6:**
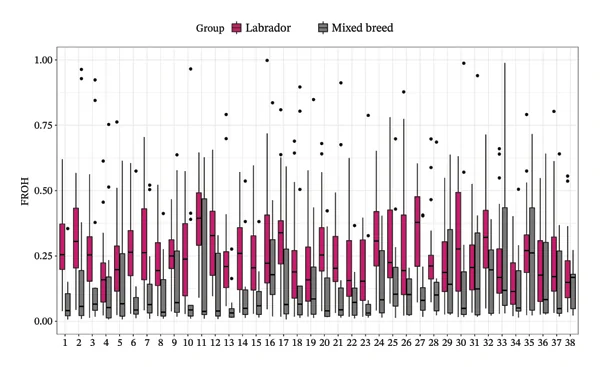
Chromosome‐wise distribution of FROH across the genome.

The distribution of FROH across autosomes (Figure 6) revealed heterogeneity among chromosomes, with certain autosomes contributing disproportionately to overall genomic inbreeding. Indeed, some autosomes, such as chromosomes 1, 2, 3, 6, 7, 13, and 27, should be investigated in terms of QTLs and selection signatures. This pattern may reflect breed‐specific selection pressures or historical bottlenecks within the Labrador population.

Altogether, ROH‐based analyses confirm higher genomic inbreeding and the presence of long autozygous segments in Labradors, aligning with expectations for a purebred population and reinforcing the relevance of integrating genomic structure into biomarker and GWAS analyses.

**FIGURE 7:**
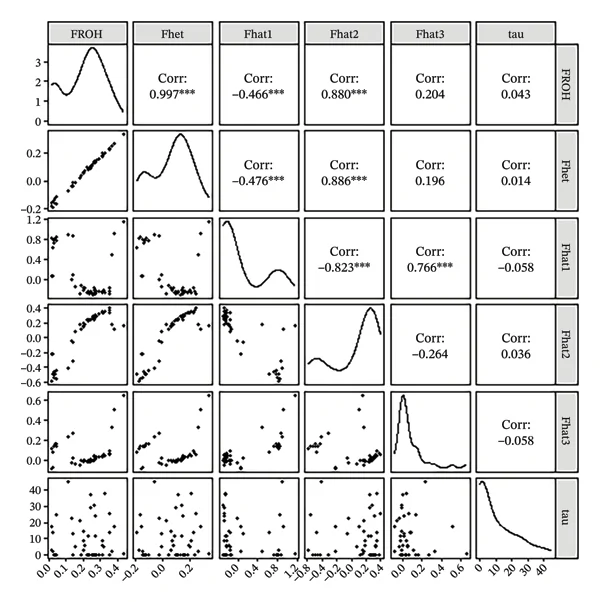
Correlation plot of genomic inbreeding estimates and pTau181 levels.

Pairwise relationships among genomic inbreeding coefficients and pTau181 are shown in Figure 7. The biomarker showed weak and nonsignificant correlations with all genomic inbreeding measures, indicating no clear linear association between pTau181 and the estimated levels of inbreeding. Scatterplots further support these findings, showing no evident trend between phosphorylated tau and any of the inbreeding coefficients.

## 4. Discussion

The present pilot study integrates peripheral molecular biomarkers and genome‐wide genetic analyses to explore interindividual variability associated with neurobiological aging in dogs. By focusing on circulating pTau181 and genomic architecture in purebred Labrador Retrievers and mixed‐breed dogs, this work provides an exploratory framework for investigating molecular and genetic contributions to aging‐related processes in a comparative context.

Circulating pTau181 was consistently detectable across all individuals, supporting the feasibility of measuring this tau‐related biomarker in canine serum, consistent with previous preliminary observations in Labrador Retrievers [36]. Mean pTau181 concentrations did not differ significantly according to breed, sex, or age category, and values showed substantial overlap across groups. This pattern suggests that peripheral pTau181 levels primarily reflect individual‐level variability rather than broad demographic factors. In humans, blood pTau181 is considered a sensitive indicator of tau‐related molecular alterations and may increase early during neurobiological aging, often preceding overt clinical manifestations [26, 27, 32, 33]. Although direct cross‐species comparisons should be made cautiously, the reliable detection of pTau181 in dogs supports its potential utility as a peripheral marker in comparative aging research.

At the molecular level, tau phosphorylation is increasingly recognized as a dynamic process occurring along a continuum from physiological aging to neurodegenerative vulnerability [18, 59]. Age‐related changes in tau phosphorylation have been reported even in the absence of overt neurodegeneration, supporting the concept that tau dysregulation may represent an early or primary component of neurobiological aging [14, 16, 17, 22]. The absence of clear age‐related differences in circulating pTau181 in this cohort may reflect the cross‐sectional design, limited sample size, and interindividual heterogeneity, rather than a lack of biological relevance.

Pedigree‐based analysis revealed very low levels of inbreeding, with only five inbred individuals and most coefficients below 0.05; a single individual exhibited an inbreeding coefficient of 0.125. An inbreeding coefficient of 0.05 is generally considered an upper acceptable threshold in dogs [60], and similarly low levels have been reported in other Italian dog populations [61]. While negative effects of inbreeding typically emerge above 5%, more pronounced fitness reductions are observed when inbreeding exceeds 10% [62, 63]. Accordingly, pedigree data suggest a low immediate risk of inbreeding depression, although even moderate inbreeding may influence phenotypic traits [64]. Owing to the limited number of inbred individuals and the narrow range of coefficients, correlations between pedigree‐based inbreeding and circulating biomarkers could not be assessed.

Although pedigree data indicated low inbreeding, genome‐wide analyses revealed substantially higher levels of autozygosity in purebred Labradors, reflected by elevated ROH and genomic inbreeding coefficients (FROH). This discrepancy is biologically informative and reflects different temporal scales: pedigree‐based measures capture recent relatedness, whereas ROH‐based metrics integrate long‐term demographic processes such as founder effects, bottlenecks, reduced effective population size, and sustained artificial selection.

Population structure analyses (admixture, PCA, and MDS) confirmed clear genetic separation between Labradors and mixed‐breed dogs, with tight clustering in Labradors indicative of reduced genetic diversity and diffuse clustering in mixed‐breeds [65, 66]. Previous work suggests that increased genomic inbreeding primarily affects disease susceptibility and age‐related traits rather than baseline biomarker levels in clinically healthy individuals [67]. Thus, the absence of population‐level differences in pTau181 does not exclude subtle, polygenic genetic influences on interindividual variability.

Purebred Labradors exhibited markedly higher ROH counts and homozygosity than mixed‐breed dogs, consistent with reduced genetic diversity resulting from long‐term artificial selection. This was reflected in a high mean FROH value (0.26), exceeding breed‐level estimates typically reported in large‐scale surveys (0.03–0.10) [65] and aligning with previous reports of increased autozygosity in purebred populations [68, 69]. The magnitude of FROH observed here likely reflects sampling effects and stringent ROH‐calling parameters, which tend to inflate ROH estimates [70]. Importantly, Labradors were not closely related, suggesting that elevated FROH values reflect historical breed‐wide processes rather than recent familial relatedness.

The ROH landscape—characterized by an enrichment of short ROH segments indicative of ancient inbreeding, alongside longer segments in some individuals suggestive of more recent relatedness—is consistent with established principles of ROH interpretation and previous studies on Labrador population history [71]. Comparisons with other purebred populations further contextualize these findings; for example, German Shorthaired Pointers show lower mean FROH values (∼0.17), suggesting relatively higher genomic diversity [72].

Increased autozygosity may have functional implications for neurobiological aging. Elevated ROH burdens have been associated with reduced fitness and increased disease susceptibility in dogs [73], supporting the hypothesis that homozygous regions may influence pathways involved in neuronal maintenance and tau metabolism. However, ROH effects are trait‐ and region‐specific, and long ROH segments do not uniformly harbor deleterious variants [74]. Thus, any impact on tau blood levels is likely to depend on the genes encompassed within specific ROH islands rather than on global ROH burden alone.

To further investigate genetic influences on pTau181 variability, we conducted an exploratory genome‐wide association analysis that identified a suggestive locus on chromosome 29 encompassing several genes of potential biological interest. Given the limited sample size and the high likelihood of false‐positive findings in GWAS, the functional annotations below should be regarded as generating hypotheses for future validation rather than as evidence of an established gene–biomarker relationship. Among the genes in this region, WWP1 encodes an E3 ubiquitin ligase involved in the ubiquitin–proteasome system, with reported roles in neuronal proteostasis and neurodegenerative disorders [75, 76] and in neuronal health and repair pathways [77]; on this basis, WWP1 may represent a biologically plausible candidate for further investigation in relation to phosphorylated tau turnover.

Other genes within this region include CPNE3, involved in neuronal development and immune responses, and ATP6V0D2, which contributes to Schwann cell–mediated myelin clearance following nerve injury [78]. Additional genes such as CNGB3, CNBD1, and RMDN1 have been linked to visual disorders, Alzheimer’s disease–related expression changes, and neuropsychiatric conditions, respectively. Several of these genes converge on pathways related to protein turnover, vesicular trafficking, and cellular stress responses, which are relevant to tau homeostasis. No clear associations were identified for PSKH2 or SLC7A13.

Several additional limitations should be acknowledged when interpreting these findings. First, the relatively small sample size limits statistical power, particularly for genome‐wide association analyses, and reduces the ability to detect modest genetic effects on circulating pTau181 levels. Replication in larger, independent canine cohorts will be required before any of the candidate genes identified here can be considered plausible contributors to pTau181 variability.

Second, the cross‐sectional design precludes inference on temporal trajectories of tau phosphorylation during aging and does not allow assessment of intra‐individual variability over time. Longitudinal studies will be essential to determine whether circulating pTau181 levels change with advancing age or precede other biological or clinical manifestations of neurobiological aging. Whether an analogous temporal pattern exists in dogs remains untested in the present cross‐sectional cohort.

Notably, studies integrating validated behavioral scales (e.g., CADES), circulating biomarkers, and postmortem neuropathological findings have demonstrated the value of multimodal approaches in dogs [79, 80] and represent an important model for future longitudinal research combining pTau181 measurement with validated cognitive and neuropathological assessment.

Additionally, cognitive status was not assessed in this cohort, using validated instruments such as the Canine Cognitive Dysfunction Rating scale or equivalent behavioral tools [81]. Consequently, the present study cannot evaluate any association between circulating pTau181 and cognitive status or decline. Whether peripheral pTau181 changes precede measurable behavioral or cognitive alterations, as suggested in some human studies [82, 83], remains untested in dogs and represents an important direction for future longitudinal, multimodal research combining biomarker and validated cognitive assessment.

Furthermore, although no overt behavioral or cognitive abnormalities were reported at the time of the general clinical examination, undetected subclinical cognitive or systemic conditions cannot be fully excluded, particularly among shelter‐housed mixed‐breed dogs, for whom clinical histories prior to enrollment were less complete than for the clinically managed Labrador Retriever cohort.

Moreover, circulating biomarkers may be influenced by peripheral factors, including metabolic turnover, renal and hepatic clearance, and blood–brain barrier permeability, which may weaken their correspondence with central nervous system processes. Consequently, peripheral pTau181 levels should be interpreted as indirect indicators of tau‐related molecular dynamics rather than direct surrogates of brain pathology. The lack of neuropathological or neuroimaging correlates further limits the ability to link peripheral tau measurements to specific brain alterations.

Finally, the heterogeneity of the study population, including differences in genetic background, environmental exposures, and life history, may contribute to interindividual variability and obscure subtle associations. While this heterogeneity reflects real‐world canine populations and enhances translational relevance, it also increases noise in exploratory analyses. Future studies combining larger, well‐characterized cohorts with standardized sampling protocols, longitudinal designs, and multimodal phenotyping will be necessary to validate and extend the present findings.

## 5. Conclusion

This pilot study explored circulating phosphorylated tau 181 in relation to genomic architecture in dogs as a comparative model of neurobiological aging. Circulating pTau181 was consistently detectable across individuals, supporting the feasibility of its measurement in canine serum. Although no associations were observed between pTau181 levels and demographic variables or global genomic inbreeding estimates, genome‐wide analyses revealed pronounced differences in genetic architecture between purebred and mixed‐breed dogs and identified suggestive genomic loci potentially contributing to interindividual variability in tau‐blood levels.

Together, these findings support the integration of peripheral tau blood levels with genome‐wide analyses as a promising exploratory approach for investigating molecular and genetic aspects of aging in dogs. Larger, longitudinal studies incorporating additional phenotypic and neuropathological data will be required to clarify the biological significance of circulating pTau181 and to validate the genetic signals identified in this study.

## Funding

Open access publishing was facilitated by Università degli Studi di Firenze, as part of the Wiley ‐ CRUI‐CARE agreement.

## Ethics Statement

The study was approved by the Ethics Committee of the University of Pisa, Italy (Delibera n. 51/2021, November 26, 2021), in accordance with Directive 2010/63/EU.

## Conflicts of Interest

The authors declare no conflicts of interest.

## Supporting Information

Additional supporting information can be found online in the Supporting Information section.

## Supporting information


**Supporting Information** Supporting Table 1: pTau181 values in each animal (Labrador and mixed breed), sex (males and females) and age (young and senior).

## Data Availability

The data presented in this study are available on request from the corresponding author.
